# Reproduction Number of the Omicron Variant Triples That of the Delta Variant

**DOI:** 10.3390/v14040821

**Published:** 2022-04-15

**Authors:** Zhanwei Du, Huaping Hong, Shuqi Wang, Lijia Ma, Caifen Liu, Yuan Bai, Dillon C. Adam, Linwei Tian, Lin Wang, Eric H. Y. Lau, Benjamin J. Cowling

**Affiliations:** 1 WHO Collaborating Centre for Infectious Disease Epidemiology and Control, School of Public Health, Li Ka Shing Faculty of Medicine, The University of Hong Kong, Hong Kong, China; zwdu@hku.hk (Z.D.); fcfliu@hku.hk (C.L.); yb424@hku.hk (Y.B.); dcadam@hku.hk (D.C.A.); linweit@hku.hk (L.T.); ehylau@hku.hk (E.H.Y.L.); 2 Laboratory of Data Discovery for Health Limited, Hong Kong Science Park, Hong Kong, China; hhp1998@d24h.hk (H.H.); crystal.sq.wang@gmail.com (S.W.); 3 College of Computer Science and Software Engineering, Shenzhen University, Shenzhen 518060, China; omegamalj@gmail.com; 4 Department of Genetics, University of Cambridge, Cambridge CB2 3EH, UK; lw660@cam.ac.uk

**Keywords:** COVID-19, SARS-CoV-2, reproduction number, Omicron, systematic review, meta-analysis

## Abstract

COVID-19 remains a persistent threat, especially with the predominant Omicron variant emerging in early 2022, presenting with high transmissibility, immune escape, and waning. There is a need to rapidly ramp up global vaccine coverage while enhancing public health and social measures. Timely and reliable estimation of the reproduction number throughout a pandemic is critical for assessing the impact of mitigation efforts and the potential need to adjust for control measures. We conducted a systematic review on the reproduction numbers of the Omicron variant and gave the pooled estimates. We identified six studies by searching PubMed, Embase, Web of Science, and Google Scholar for articles published between 1 January 2020 and 6 March 2022. We estimate that the effective reproduction number ranges from 2.43 to 5.11, with a pooled estimate of 4.20 (95% CI: 2.05, 6.35). The Omicron variant has an effective reproduction number which is triple (2.71 (95% CI: 1.86, 3.56)) that of the Delta variant.

## 1. Introduction

COVID-19 remains a persistent threat, especially with the predominant Omicron variant emerging in early 2022, presenting with high transmissibility, immune escape, and waning. There is a need to rapidly ramp up global vaccine coverage while enhancing public health and social measures. Timely and reliable estimation of the reproduction number throughout a pandemic is critical for assessing the impact of mitigation efforts and the potential need to adjust for control measures.

Five SARS-CoV-2 variants of concern (VOC)—the Alpha, Beta, Gamma, Delta, and Omicron variants—have been identified as of 6 March 2022 [1]. The most recent, Omicron, was first identified in November 2021 and has since been reported in 194 countries and territories [2]. The basic reproduction number (*R*_0_) and the effective reproduction number (*R_e_*) are two key epidemiological metrics used to (i) estimate the average number of secondary infections caused by each infected case in a fully susceptible population and (ii) estimate the average number of secondary infections at a given time point during an epidemic, respectively. Accurate estimates of *R*_0_ and *Re* of SARS-CoV-2 variants are essential for tracking pandemic progress, and for determining the need for and strength of control measures, such as social distancing, to mitigate transmission. Here, we performed a systematic review and meta-analysis of the published estimates of *R*_0_ and *R_e_* for the SARS-CoV-2 Omicron variant.

## 2. Methods

### 2.1. Search Strategy and Selection Criteria

All searches were carried out on 6 March 2022 in PubMed, Embase, Web of Science, and Google Scholar for articles published from 1 January 2020 to 6 March 2022. Our search terms for reproduction numbers of SARS-CoV-2 variant included (#1) “COVID-19” or “SARS-CoV-2” or “2019-nCoV” or “coronavirus”; (#2) “reproduct* number” or “reproduct* ratio” or “reproduct* rate” or “transmissibility”; and (#3) Omicron or “B.1.1.529”. Our final search term was #1 and #2 and #3. After reading the abstract and full text, we included the studies that provided information about the estimated reproduction numbers of the Omicron variant and the relative ratio of the Omicron variant to the Delta variant.

### 2.2. Data Extraction

All data were extracted independently and entered in a standardized form by 3 co-authors (Z.D., H.H., and S.W.). Conflicts over inclusion of the studies and retrieving the estimates of these variables were resolved by Z.D. Information was extracted on the estimates of reproduction number of COVID-19 variants coupled with the corresponding 95% confidence interval (CI) or the 95% credible interval (CrI). Other information, such as the study location and the estimation methods, were also extracted for each selected study. For studies that estimated the ratio, $R_{e}/R_{\delta }{}^{}$, in the reproduction number, for the Omicron variant versus the Delta variant, we calculated the effective reproduction number of the Omicron variant by adopting the values of $R_{e}$ of the Delta variant, estimated from other studies.

### 2.3. Statistical Analysis

A random effects model was further used to perform a meta-analysis in this study. Analyses were conducted in R version 4.1.1 (R Foundation for Statistical Computing, Vienna, Austria). Specifically, for studies providing the mean and with 95% confidence intervals, we use R function metagen of the meta package to estimate the pooled estimates of effective reproduction numbers using the random effects model. Our R codes are publicly available upon publication at github (https://github.com/ZhanweiDU/R_Omicron/, accessed on 10 April 2022).

## 3. Results

**Figure 1:**
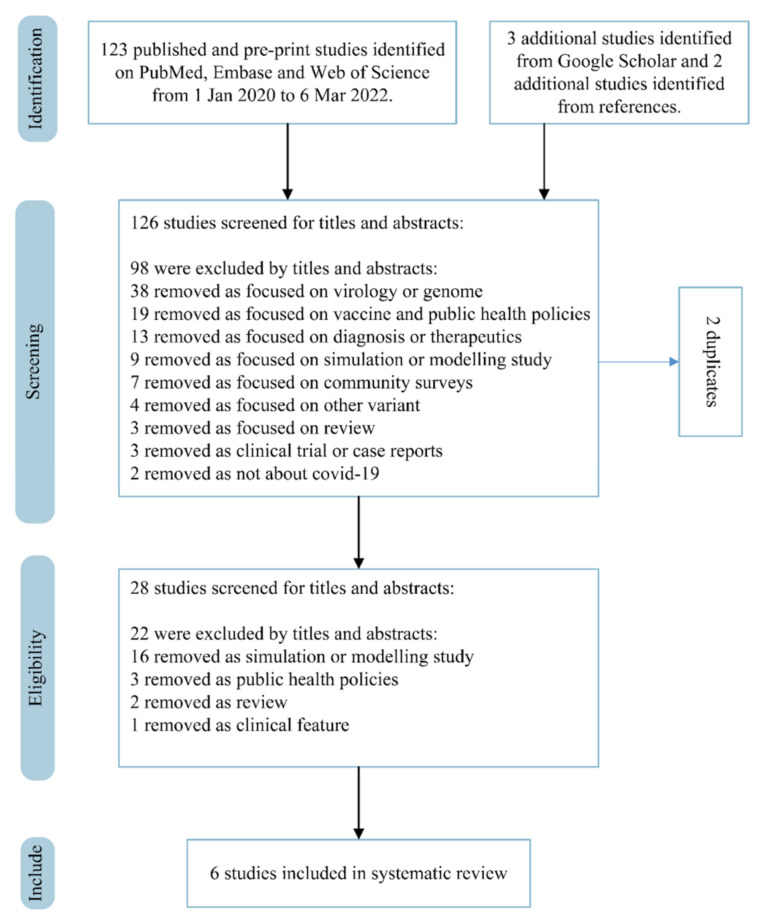
PRISMA (Preferred Reporting Items for Systematic Reviews and Meta-Analyses) flow diagram for the studies used to obtain studies that reported reproduction numbers.

We identified 123 studies by searching PubMed, Embase, Web of Science, and Google Scholar for articles published between 1 January 2020 and 6 March 2022, and additionally included 3 studies from our own reference list. Of these, 2 duplicates were removed, and 98 studies were excluded through title and abstract screening, leaving 28 studies for full-text assessment. Six of them were finally included in this study, providing one *R*_0_ estimate and six *Re* estimates. The detailed selection process is illustrated in Figure 1. The reported substrains include BA.1, BA.2, and BA.1.1, among which BA.1 was the most studied. The final six selected studies were conducted in South Africa, Denmark, South Africa, China, England, and India, respectively (Table 1).

Estimates of reproduction numbers were reported for South Africa, Denmark, China, England, and India, with mean estimates of Re ranging from 2.43 to 5.11 (Table 1 and Table S1), together with the reported reproduction numbers of the Delta variant over countries [2]). After excluding one study [8] where the 95% CI was not reported, the pooled estimate of Re was 4.20 (95% CI: 2.05, 6.35). Overall, the Omicron variant has a higher reproduction number than that of the Delta variant. The ratio of the reproduction numbers between the Omicron and the Delta variant ranges from 1.60 to 4.20, with a pooled estimate of 2.71 (95% CI: 1.86, 3.56), denoting that the Omicron variant has a higher transmission potential compared with other earlier variants (Alpha, Beta, Delta, Epsilon, Eta, Gamma, Iota, Kappa, Zeta, R.1 (a Pango lineage not labeled by the WHO), B.1.1.519, B.1.1.222, N501Y, and D514G (lineages not labeled by the WHO)) [10].

## 4. Discussion

Given the ongoing appearance of new variations, the pandemic’s future is bleak [11]. Throughout the pandemic, governments resorted mostly to mass vaccination to decrease transmission and reduce mortality [12]. To prevent the spread of variants with a higher transmissibility, more drastic interventions may be required. It is thus necessary to guide control measures in reducing the propagation of viruses by accurately estimating the reproduction number for various wild-type genetic variants. This systematic review may provide epidemiologists with practical guidelines of the range of effective reproduction numbers across countries to fit transmission rates in epidemic simulation, perhaps for estimates of importation risks from regions with Omicron outbreaks to a susceptible region. As more specific estimates for Omicron substrains are available, further updates should be made to inform epidemic simulation.

A few caveats need to be discussed. First, we did not include factors potentially correlated with estimates of the reproduction number, such as contact patterns and climatic factors, because of data availability. Second, we only studied basic and effective reproduction numbers to assess the transmissibility of SARS-CoV-2 variants, rather than that of transmission advantage using other metrics apart from reproduction numbers. Third, most of the eligible studies in our review did not account for waning of immunity and re-infection, which could impact the comparison of effective reproduction numbers. Fourth, some studies estimate reproductive numbers in the early period of outbreaks, which may provide lower values. We use the reported estimates of reproduction numbers directly without any adjustment. Fifth, there are three substrains (e.g., BA.1, BA.2, and BA.1.1) of the Omicron variant in our studies. BA.2 is expected to be more transmissible than BA.1 [13]. The effective reproduction number in the study period with the substrain mixing of BA.2 and others may be underestimated when BA.2 has been the prevalent Omicron substrain globally since January 2022 [14].

In conclusion, multiple estimates of the effective reproduction number of the Omicron variant have been reported, tripling that of the Delta variant. COVID-19 remains a persistent threat, especially with the predominant Omicron variant presenting in early 2022 with high transmissibility, immune escape, and waning. There is a need to rapidly ramp up global vaccine coverage while enhancing public health and social measures. 

## Figures and Tables

**Table 1:** Description of studies included for the Omicron variant of SARS-CoV-2.

Study	$R_{e}$ (Mean and 95% CI)	$R_{e}/R_{\delta }{}^{}$ (Mean and 95% CI)	Period	Country	Substrain
[3]	5.11 (4.30–6.01)	3.31 (2.95–3.72)	January to December 2021	South Africa	BA.1 [4]
[5]	4.93 (4.13–5.82)	3.19 (2.82–3.61)	November 2021 to January 2022	Denmark	BA.1, BA.2, BA.1.1 [4]
[6]	7.57 (4.12–12.70)	4.20 (2.10–9.10)	September to November 2021	South Africa	BA.1 [4]
[7]	2.43 (1.05–5.49)	1.74 (0.66–4.86)	January 2022	China	BA.1
[8]	4.0	3.05 (2.35–4.82)	December 2021 to April 2022	England	BA.1, BA.2, BA.1.1 [4]
[9]	2.57 (1.34–3.57)	1.60 (1.02–2.26)	December 2021 to January 2022	India	BA.1, BA.2, BA.1.1 [4]

$R_{e}/R_{\delta }{}^{}$ denotes the ratio of effective reproduction numbers between the Omicron variant (*Re*) with the Delta variant ($R_{\delta }$). More details can be found in Table S1.

## Data Availability

All data are collected from open source with detailed description in Supplementary Materials.

## Supplementary Materials

The following supporting information can be downloaded at: https://www.mdpi.com/article/10.3390/v14040821/s1, Table S1. Description of studies included for the Omicron variant of SARS-CoV-2. References [3,4,5,6,7,8,9,10] are cited in the supplementary materials.

## References

[1] Tracking SARS-CoV-2 Variants. https://www.who.int/en/activities/tracking-SARS-CoV-2-variants/.

[2] Statista (2021). Variants of Concern SARS-CoV-2 Comparison. https://www.statista.com/statistics/1206597/coronavirus-new-variants-comparison/.

[3] Suzuki R., Yamasoba D., Kimura I., Wang L., Kishimoto M., Ito J., Morioka Y., Nao N., Nasser H., Uriu K. (2022). Attenuated fusogenicity and pathogenicity of SARS-CoV-2 Omicron variant. Nature.

[4] GISAID hCov19 Variants. https://www.gisaid.org/hcov19-variants/.

[5] Ito K., Piantham C., Nishiura H. (2021). Relative instantaneous reproduction number of Omicron SARS-CoV-2 variant with respect to the Delta variant in Denmark. J. Med. Virol..

[6] Nishiura H., Ito K., Anzai A., Kobayashi T., Piantham C., Rodríguez-Morales A.J. (2021). Relative Reproduction Number of SARS-CoV-2 Omicron (B.1.1.529) Compared with Delta Variant in South Africa. J. Clin. Med. Res..

[7] Ruan F., Zhang X., Xiao S., Ni X., Yin X., Ye Z., Chen G., Zhu T., Chen Z., Yao G. (2022). An Outbreak of the COVID-19 Omicron Variant—Zhuhai City, Guangdong Province, China, January 13, 2022. China CDC.

[8] Barnard R.C., Davies N.G., Pearson C.A.B., Jit M., John Edmunds W. (2022). Projected epidemiological consequences of the Omicron SARS-CoV-2 variant in England, December 2021 to April 2022. medRxiv.

[9] Ranjan R. (2022). Omicron impact in India: Analysis of the ongoing COVID-19 third wave based on global data. medRxiv.

[10] Du Z., Liu C., Wang C., Xu L., Xu M., Wang L., Bai Y., Xu X., Lau E.H.Y., Wu P. (2022). Reproduction Numbers of SARS-CoV-2 Variants: A Systematic Review and Meta-analysis. Clin. Infect. Dis..

[11] Wang Z., Schmidt F., Weisblum Y., Muecksch F., Barnes C.O., Finkin S., Schaefer-Babajew D., Cipolla M., Gaebler C., Lieberman J.A. (2021). mRNA vaccine-elicited antibodies to SARS-CoV-2 and circulating variants. Nature.

[12] Lai S., Ruktanonchai N.W., Zhou L., Prosper O., Luo W., Floyd J.R., Wesolowski A., Santillana M., Zhang C., Du X. (2020). Effect of non-pharmaceutical interventions to contain COVID-19 in China. Nature.

[13] Statement on Omicron Sublineage BA.2. https://www.who.int/news/item/22-02-2022-statement-on-omicron-sublineage-ba.2.

[14] Callaway E. (2022). Why does the Omicron sub-variant spread faster than the original?. Nature.

