# Supplementary material for: Reproduction Number of the Omicron Variant Triples That of the Delta Variant
Source: Viruses. 2022 Apr 15;14(4):821. doi: 10.3390/v14040821 (PMC9027795; doi:10.3390/v14040821)
Supplement: Supplementary file 1 [file viruses-14-00821-s001.zip › viruses-1657773-supplementary.pdf]

viruses

MDPI

Supplementary Materials

Reproduction Number of the Omicron Variant Triples That of the Delta Variant

Table S1. Description of studies included for the Omicron variant of SARS-CoV-2.

Study	R_0 (mean and 95% CI)	R_e (mean and 95% CI)	R_e/R_δ (mean and 95% CI)	R_δ	Period	Region	Method	Substrain
[3]		5.11~ (4.30-6.01)	3.31* (2.95-3.72)	1.54 (95% CI, 1.27-1.81), as the pooled estimate over regions [10]	2021-01-01 2021-12-24	South Africa	Bayesian statistical model	BA.1 [4]
[5]		4.93~ (4.13-5.82)	3.19 (2.82-3.61)	1.54 (95% CI, 1.27-1.81), as the pooled estimate over regions [10]	2021-11-01 2022-01-08	Denmark	Maximum likelihood method	BA.1, BA.2, BA.1.1 [4]
[6]		7.57~ (4.12-12.7)	4.20 (2.10-9.10)	1.54 (95% CI, 1.27-1.81), as the pooled estimate over regions [10]	2021-09-23 2021-11-18	Gauteng, South Africa	Maximum likelihood method	BA.1 [4]
[7]	1.3 (0.8-2.0)	2.43 (1.05-5.49)	1.74# (0.66-4.86)	1.24 (95% CI, 0.47-3.52) in China [10]	2022-01-12 2022-01-21	Zhuhai, China	Maximum likelihood method	BA.1
[8]		4.0	3.05# (2.35-4.82)	1.39 (95% CI, 0.69-1.81) in England [10]	2021-12-01 2022-04-30	England	Exponential growth rate method	BA.1, BA.2 , BA.1.1 [4]
[9]		2.57 (1.34-3.57)	1.60# (1.02-2.26)	1.54 (95% CI, 1.27-1.81), as the pooled estimate over regions [10]	2021-12-28 2022-01-10	India	Maximum likelihood method	BA.1, BA.2, BA.1.1 [4]

Note: * The reproduction number is reported by median and 95% credible interval (CrI); ~ We assume that both R_δ for the Delta variant and relative change R_e/R_δ follow triangular distributions. We assume the lower limit, upper limit, and mode of the triangular distribution as the lower and upper 95% CI and the mean estimates, respectively. We generate 10,000 pairs based on triangular distributions of reproduction number for both R_e and R_e/R_δ , respectively. By multiplying R_δ and R_e/R_δ in each pair, we can estimate R_e for the Omicron variant and conduct the statistics analysis; # We assume that effective reproduction numbers of R_δ for both the Delta variant and R_δ for the Omicron variant follow triangular distributions. We assume the lower limit, upper limit, and mode of the triangular distribution as the lower and upper 95% CI and the mean

estimates, respectively. We generate 10,000 pairs based on triangular distributions of reproduction number for both R_e and $\boldsymbol{R_e/R_\delta}$, respectively. By dividing R_e into R_e/R_δ in each pair, we can estimate R_e for the Omicron variant.
